# Magnetic Resonance Thrombus Imaging to Differentiate Acute from Chronic Portal Vein Thrombosis

**DOI:** 10.1055/s-0040-1716716

**Published:** 2020-09-23

**Authors:** Lisette F. van Dam, Frederikus A. Klok, Maarten E. Tushuizen, Walter Ageno, Sarwa Darwish Murad, Guido R. van Haren, Menno V. Huisman, Mandy N. Lauw, Antonio Iglesias del Sol, Martin N.J.M. Wasser, Ysbrand Willink, Lucia J.M. Kroft

**Affiliations:** 1 Department of Thrombosis and Hemostasis, Leiden University Medical Center, Leiden, The Netherlands; 2 Department of Gastroenterology and Hepatology, Leiden University Medical Center, Leiden, The Netherlands; 3 Department of Medicine and Surgery, University of Insubria, Varese, Italy; 4 Department of Gastroenterology and Hepatology, Erasmus University Medical Center, Rotterdam, The Netherlands; 5 Department of Radiology, Leiden University Medical Center, Leiden, The Netherlands; 6 Department of Hematology, Erasmus University Medical Center, Rotterdam, The Netherlands; 7 Department of Internal Medicine, Alrijne Hospital, Leiderdorp, The Netherlands

**Keywords:** diagnosis, diagnostic imaging, magnetic resonance imaging, venous thrombosis

## Abstract

**Introduction** Timely diagnosis and treatment of portal vein thrombosis (PVT) is crucial to prevent morbidity and mortality. However, current imaging tests cannot always accurately differentiate acute from chronic (nonocclusive) PVT. Magnetic resonance noncontrast thrombus imaging (MR-NCTI) has been shown to accurately differentiate acute from chronic venous thrombosis at other locations and may also be of value in the diagnostic management of PVT. This study describes the first phase of the Rhea study (NTR 7061). Our aim was to select and optimize MR-NCTI sequences that would be accurate for differentiation of acute from chronic PVT.

**Study Design** The literature was searched for different MRI sequences for portal vein and acute thrombosis imaging. The most promising sequences were tested in a healthy volunteer followed by one patient with acute PVT and two patients with chronic PVT, all diagnosed on (repetitive) contrast-enhanced computed tomography (CT) venography to optimize the MR-NCTI sequences. All images were evaluated by an expert panel.

**Results** Several MR-NCTI sequences were identified and tested. Differentiation of acute from chronic PVT was achieved with 3D T1 TFE (three-dimensional T1 turbo field echo) and 3D T1 Dixon FFE (three-dimensional T1 fast field echo) sequences with best image quality. The expert panel was able to confirm the diagnosis of acute PVT on the combined two MR-NCTI sequences and to exclude acute PVT in the two patients with chronic PVT.

**Conclusion** Using 3D T1 TFE and 3D T1 Dixon FFE sequences, we were able to distinguish acute from chronic PVT. This clinical relevant finding will be elucidated in clinical studies to establish their test performance.

## Introduction

Acute portal vein thrombosis (PVT) has a poor short- and long-term prognosis 1 and a higher mortality rate than the “usual site” venous thromboembolism, that is, acute pulmonary embolism and deep vein thrombosis (DVT). Therefore, timely diagnosis and anticoagulant treatment are crucial for the patients' prognosis. 2 3 4 Distinguishing between a fresh thrombus, in which anticoagulant treatment is indicated, and a chronic (organized, nonresolvable) thrombus is of paramount importance for treatment decision. 5 However, currently available imaging tests are not always able to accurately differentiate acute from chronic PVT, especially when it concerns an organized nonocclusive chronic thrombus without signs of cicatrization of the affected vessel.

Magnetic resonance noncontrast thrombus imaging (MR-NCTI), also referred to as magnetic resonance direct thrombus imaging (MRDTI) in previous publications, is a noncontrast-enhanced magnetic resonance imaging (MRI) technique, which allows for direct visualization of acute thrombi. 6 This technique is based on the formation of methemoglobin in a fresh thrombus, which appears as high signal intensity on a T1-weighted MRI sequence by shortening the T1 relaxation time ( Supplementary Fig. 1 ). 6 This increased signal intensity fades when the thrombus ages over a period of 3 to 6 months. 7 MR-NCTI has been shown to accurately differentiate acute from chronic DVT and to safely exclude acute recurrent ipsilateral DVT of the legs. 7 8 9 The diagnostic accuracy of MR-NCTI has not yet been evaluated for the diagnosis of PVT. Because imaging of abdominal veins differs in many ways from imaging of veins in the extremities, for example, artifacts from ascites and intestinal movement and gas, the MR-NCTI sequences used in the extremities need to be adjusted for optimal visualization of PVT. We set out to select and optimize MR-NCTI sequences for differentiation between acute and chronic PVT.

**Fig. 1:**
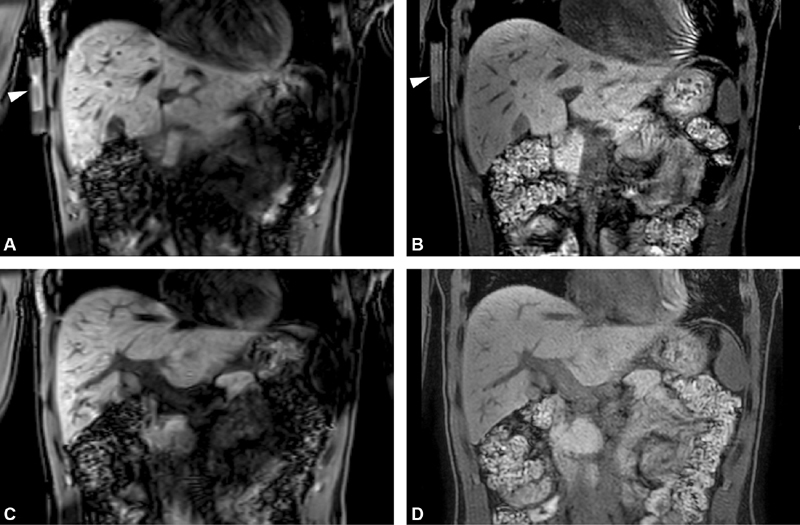
( **A–D** ) Magnetic resonance (MR) images of the abdomen in coronal view of a healthy volunteer with a coagulation tube attached to the abdomen. ( **A, C** ) 3D T1 TFE (three-dimensional T1 turbo field echo) images at two levels showing a low signal intensity in intrahepatic veins and good contrast resolution between the liver parenchyma and veins. High signal intensity is present in the coagulation tube with clotted blood (arrow). ( **B,D** ) 3D T1 Dixon FFE (three-dimensional T1 fast field echo sequence) (water-only) images at two levels showing a low signal intensity in the intrahepatic veins and intermediate signal intensity in the coagulation tube (arrow).

## Materials and Methods

This study is the first phase of the Rhea study (NTR 7061), a prospective diagnostic study to evaluate the diagnostic accuracy of MR-NCTI for distinguishing acute from chronic PVT. In the Rhea study, our aim is to include 70 PVT patients, including 35 patients with confirmed acute PVT (partial or occlusive), that is, acute symptoms (<2 weeks) characteristic for PVT, diagnosed with Doppler ultrasound (Doppler US), computed tomography (CT) venography, or MRI, and 35 patients with confirmed nonsymptomatic chronic PVT (partial or occlusive), that is, unchanged chronic thrombi on two serial imaging tests with an at least 3-month interval. The study protocol and its amendments were approved by the Institutional Review Board of the Leiden University Medical Center, Leiden, the Netherlands, and the University of Insubria, Varese, Italy. All patients will be asked for written informed consent.

For this first phase of the Rhea study, we aimed to select and develop MR-NCTI sequences that would be accurate for differentiation of acute from chronic PVT. First, a literature search for different MRI sequences for portal vein and acute thrombosis imaging was performed. The literature search was conducted in PubMed for papers published in English and in humans on December 16, 2019 (search strategy detailed in Supplementary Material ). Because imaging of the abdomen is hampered by intestinal movements and gas, search terms included techniques with correction for respiratory motion artifacts, good spatial and contrast resolution, and fat suppression.

The most promising sequences were adjusted for abdominal imaging and tested in a healthy volunteer using an MRI 3.0-Tesla unit (Philips Ingenia, Philips Medical Systems, Best, the Netherlands). A 55-cm receive-only body multicoil (combination of posterior and anterior coils) was used. Image assessment involved acquiring images in the coronal and axial planes with standard image reconstruction techniques. To gain knowledge of the performance of the sequence to detect fresh blood clots in addition to the visualization of the venous anatomy, two tests coagulation tubes were attached to the abdomen of the volunteer: one was filled with water (control) and one with clotted blood of a healthy volunteer, prepared and stored at room temperature at least 48 hours before each scanning session. The test sequence scan parameters were adjusted until adequate image quality of the veins with high signal intensity for thrombus in the coagulation tube was achieved.

MR-NCTI sequences showing the best image quality and contrast resolution were tested in three patients with confirmed PVT, including one patient with acute PVT and two patients with chronic PVT. Scan optimization was performed until a clear distinct signal intensity was achieved between acute and chronic PVT.

Finally, the MRI images of the three PVT patients were evaluated by an expert panel consisting of two radiologists (L.K. and M.W.) with over 20 years of experience with vascular MRI, one radiology technician (G.H.) with over 20 years of experience with vascular MRI acquisition, one internist (F.K.) with 6 years of experience, and one researcher (L.D.) with 2 years of experience with vascular MRI interpretation. The evaluation of the scans was performed and compared with the clinical presentation and the results of other imaging (Doppler US, CT venography, or MRI). The scan results were assessed for image quality, venous location, and either the presence or absence of acute thrombosis.

## Results

### Literature Search

We identified the following MRI sequences: three-dimensional T1 turbo field echo (3D T1 TFE), 3D turbo spin echo with spectral attenuated inversion recovery (3D TSE SPAIR), and T1 high-resolution isotropic volume excitation (THRIVE) and techniques: black-blood, Dixon and Principle of Selective Excitation Technique (ProSET) which could be suitable for portal vein imaging.

A 3D T1 TFE sequence was shown to be highly accurate for the diagnosis of first DVT and the differentiation of acute DVT from chronic residual vascular abnormalities in the leg. 8 9 10 Hence, this sequence seemed promising for the differentiation of acute from chronic PVT. Furthermore, in a pilot study, 3D T1 TFE and 3D TSE SPAIR sequences successfully confirmed the diagnosis of arm vein thrombosis when compared with ultrasonography or contrast venography. 11 The advantages of 3D TSE-SPAIR over 3D T1 TFE were a higher spatial resolution of the vessel wall and less high signal artifacts in arteries caused by inflow effects. Therefore, both 3D T1 TFE and 3D TSE SPAIR may be suitable for portal vein imaging.

THRIVE sequences may also be a promising MR-NCTI technique in PVT with potential good contrast resolution between high signal intensity of an acute thrombus and low signal intensity intravascular. 12

The black-blood technique was successfully used to identify DVT of the leg and in cerebral vein thrombosis. 13 14 With this technique, the signal from flowing blood is nullified and highlights static anatomy including thrombi. 15 Because of known good contrast resolution between thrombus and flowing blood, we hypothesized that combining the black-blood technique with 3D TSE SPAIR may be suitable for portal vein imaging.

Since it can be difficult to distinguish an acute thrombus from other tissues with a short T1 relaxation time, for example, fat tissue, which is present in the liver, adding a fat-suppression technique that suppresses the fat signal may help in depicting thrombosis. With this technique, selective pulses cause signal from fat to be nulled (saturated), whereas the water signal is relatively unaffected. The Dixon technique is an often-used fat suppression technique that can be used with several sequences such as T1- and T2-weighted imaging and gradient echo MRI. 16 17 An alternative to fat suppression methods is water excitation by means of a spectral spatial pulse. With this technique, only water is excited by using section-selective composite pulses, whereas lipid spins are left in equilibrium, thereby producing no signal. 18 ProSET, a water excitation technique, was successfully used in a pregnant patient with proximal iliac vein thrombosis 19 and may therefore be applicable in portal vein imaging as well.

### Sequence Testing and Optimization

All three selected imaging sequences (3D T1 TFE, 3D TSE SPAIR, and THRIVE), and the black-blood, Dixon, and ProSET techniques were tested in a healthy volunteer.

3D T1 TFE sequence combined with the ProSET technique and the Dixon technique added to a 3D T1 Dixon FFE showed good contrast resolution between the liver parenchyma and intrahepatic veins. Moreover, a high signal intensity was acquired from the coagulation tube ( Fig. 1 ). These two sequences were thus the most promising for portal vein imaging and were evaluated in three PVT patients ( Table 1 ).

**Table 1:** 3D T1 TFE and T1 Dixon FFE scan parameters

	3D T1 TFE	3D T1 Dixon FFE
Technique	T1 TFE	T1 FFE
Orientation	Coronal	Coronal/Transversal
Respiratory motion suppression	Respiratory gating	Breath hold
Slices	80	160
Slice thickness (mm)	4.0	3.0
Slice distance (mm)	2.0	1.5
FOV	400
Voxel size (mm)	1.56 × 2.24 × 4	1.7 × 1.7 × 3.5
Scan time (ms)	02:36	00:19
Echo time (ms)	3.73	−
Repetition time (ms)	7.41	3.75
Flip angle	10	15

Abbreviations: 3D T1 Dixon FFE, three-dimensional T1 fast field echo sequence; 3D T1 TFE, three-dimensional T1 turbo field echo; FOV, field of view.

3D TSE SPAIR, with and without the black-blood technique, and THRIVE sequence were shown to be suboptimal for abdominal direct thrombus imaging because of a high signal intensity from blood flow in splanchnic veins and intestines despite saturation slab and low image quality due to motion artifacts. These sequences were excluded for further analysis.

### MR-NCTI Optimization in PVT Patients

**Fig. 2:**
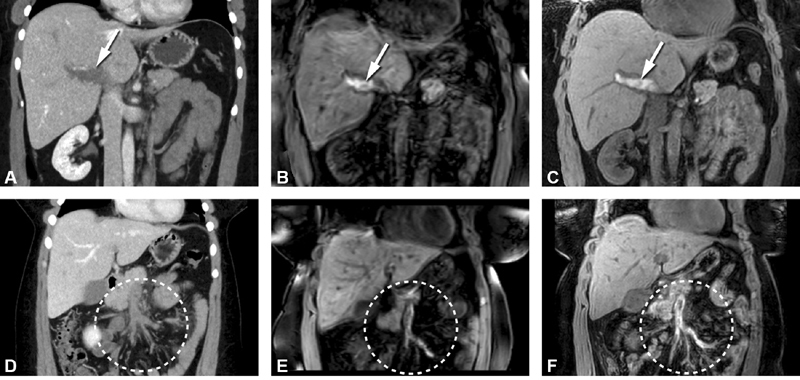
( **A–F** ) Coronal computed tomography (CT) and magnetic resonance imaging (MRI) of the abdomen of a patient diagnosed with acute thrombosis in portal and mesenteric veins. ( **A** ) CT image after intravenous contrast administration in portal-venous contrast phase shows a large luminal filling defect in the portal vein (arrow). ( **B** ) MRI 3D T1 TFE (three-dimensional T1 turbo field echo) image shows a high signal intensity in the portal vein compatible with acute thrombus (arrow). ( **C** ) MRI, 3D T1 Dixon FFE (three-dimensional T1 fast field echo sequence) (water-only) image shows a high signal intensity in the portal vein (arrow). ( **D** ) CT image after intravenous contrast administration shows extensive filling defects in mesenteric veins with increased attenuation of the surrounding mesenteric fat (encircled). ( **E** ) MRI 3D T1 TFE image shows a high signal intensity in the mesenteric veins compatible with acute thrombus (encircled). ( **F** ) MRI 3D T1 Dixon FFE (water-only) image shows a high signal intensity in the mesenteric veins (encircled). Note: acute portal vein thrombosis (PTV) is defined as an acute onset (<2 weeks' existent) of symptoms characteristic for PVT (including but not limited to abdominal pain) diagnosed with Doppler ultrasound, CT venography, or MRI.

**Fig. 3:**
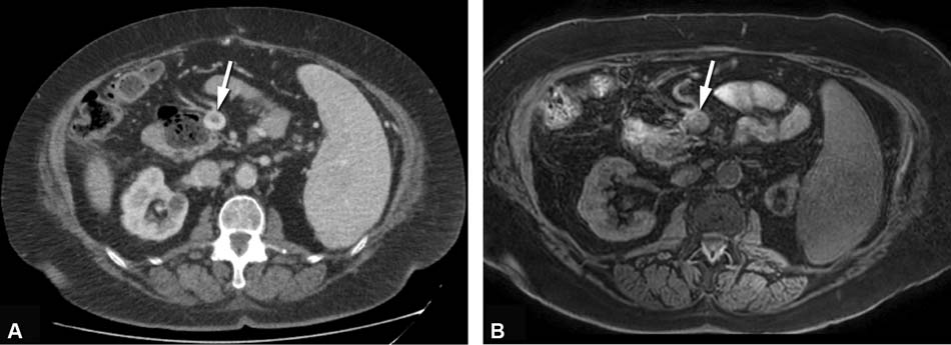
( **A** ) Computed tomography (CT) axial image of the abdomen in portal-venous contrast phase after intravenous contrast administration showing a central luminal contrast defect caused by chronic thrombus in the mesenteric vein (arrow). ( **B** ) Magnetic resonance imaging (MRI) axial 3D T1 Dixon FFE (three-dimensional T1 fast field echo sequence) image of the same patient showing isointense signal intensity in the mesenteric vein (arrow). On MRI, the signal intensity of the filling defect in the mesenteric vein is not increased, indicating chronic thrombus. Note: Chronic thrombosis is defined as nonsymptomatic chronic thrombosis defined by unchanged chronic thrombi on two serial imaging tests with an at least 3-month interval.

3D T1 TFE and 3D T1 Dixon FFE sequences were used to evaluate one patient with acute PVT and two patients with chronic PVT ( Table 2 ). 3D T1 TFE and 3D T1 Dixon FFE showed a high signal intensity in all abdominal vein segments with acute thrombosis diagnosed on CT venography ( Fig. 2 ). In the two patients with chronic PVT, both sequences showed no increased signal intensity in the portal or mesenteric veins ( Fig. 3 ). The expert panel was able to confirm the diagnosis of acute PVT on the combined two MR-NCTI sequences and to exclude acute PVT in the two patients with chronic thrombosis. The combination of 3D T1 TFE and 3D T1 Dixon FF was thus judged optimal for locating and differentiating acute from chronic PVT with good image quality and short scanning time (10–15 minutes).

**Table 2:** Patient characteristics of the three patients diagnosed with PVT

	Diagnosis at inclusion	Sex	Age (y)	Relevant patient history	Previous VTE?	Anticoagulants at inclusion	Risk factors for VTE
Patient 1	Acute PVT	Female	35	None	No	Hormonal contraception use
Patient 2	Chronic PVT	Male	29	Myeloproliferative neoplasia complicated by thrombosis of the mesenteric, portal, and splenic vein. Portal hypertension with esophageal varices	Yes	No	Myeloproliferative neoplasia ( *JAK2* mutation)
Patient 3	Chronic PVT	Female	67	NASH liver cirrhosis for which liver transplantation. Thrombosis of the superior mesenteric vein with ischemic small bowel for which ileum resection	Yes	NASH liver cirrhosis

Abbreviations: NASH, nonalcoholic steatohepatitis; PVT, portal vein thrombosis; VTE, venous thromboembolism; *JAK2* , janus kinase 2.

Note: Acute PVT is defined as an acute onset (<2 weeks' existent) of symptoms characteristic for PVT, diagnosed with Doppler ultrasound, computed tomography venography, or magnetic resonance imaging. Chronic PVT is defined as nonsymptomatic chronic PVT defined by unchanged chronic thrombi on two serial imaging tests with an at least 3-month interval.

## Discussion

In this first phase of the Rhea study, the combination of 3D T1 TFE and 3D T1 Dixon FFE sequences was found to be the most optimal for diagnosing and differentiating acute from chronic PVT. Differentiation between acute and chronic PVT is essential as current guidelines recommend different anticoagulant strategies in patients with acute or chronic PVT, though based on (very) low level of evidence. 5 However, with currently available imaging tests, Doppler US, CT venography, and MRI, it is not always possible to accurately differentiate acute from chronic thrombosis. The diagnosis of chronic PVT can be made in cases of morphologic changes such as an atretic portal vein, inflow branches, and cavernous transformation of the portal vein, also called portal cavernoma. 20 21 However, it may be impossible to differentiate acute from chronic PVT in the presence of an organized nonocclusive chronic PVT, without these morphological changes. Furthermore, up to 30% of the PVT cases are detected incidentally in imaging studies performed for other indications in which it is very challenging to determine whether the incidentally observed thrombosis is acute, chronic, or even an imaging artifact. 22

MR-NCTI has been shown to be a valuable diagnostic test for the diagnosis of upper extremity DVT, pelvic vein, and cerebral vein thrombosis, 11 14 19 and was shown to be an accurate, simple, feasible, and reproducible diagnostic test in suspected recurrent ipsilateral DVT of the leg. 9 There are some important differences between venous thrombosis of the portal vein and that at other locations. For instance, portal vein imaging is different from imaging of veins in the extremities and brain due to the presence of ascites, gas, and bowel movements, which can hamper abdominal vein imaging. Furthermore, PVT occurs in different clinical circumstances (i.e., portal hypertension, cirrhosis) that are less relevant to typical VTE. 23

There are only a few case reports available on noncontrast-enhanced MRI for the diagnosis of PVT using different techniques and without results on the diagnostic accuracy of MRI for PVT. 24 25 In a study by Zirinsky et al, T1- and T2-weighted MR images of 14 patients with acute and chronic PVT on CT or ultrasonography and of 8 patients with portal hypertension but without evidence of PVT were evaluated. With MRI, (sub-)acute (<5 weeks old) thrombi appeared hyperintense relative to liver and muscle on both T1- and T2-weighted sequences, and older thrombi (2–18 months old) appeared hypertense in some patients but only on T2-weighted images. Therefore, it was suggested that chronic thrombosis may lose its relative hyperintensity on T1-weighted images and thus may be used for detecting and classification of PVT. 24 In a case series by Haddad et al, thrombosis on noncontrast-enhanced T1-weighted images appeared different depending on the age of the thrombus, with a high signal intensity in subacute splanchnic vein thrombosis (<6 weeks) and low signal intensity in more chronic (>2 months old) thrombosis. 25

There are some limitations of this study. First, this study includes only three patients and therefore the validity of the results must be evaluated in a large cohort before we can proceed to an outcome study. Such an accuracy study is currently ongoing. Furthermore, the evaluation of MR-NTCI images by the expert panel was not blinded for other imaging studies including ultrasound and CT images. Additionally, due to the small number of patients, the interobserver agreement could not be established.

In conclusion, we were able to identify two MR-NTCI sequences, 3D T1 TFE and 3D T1 Dixon FFE, that were able to diagnose and differentiate acute from chronic PVT. With our previous experience based on imaging of DVT in the lower and upper extremities, 9 11 19 we believe that the image quality is sufficient to be of clinical value and initiate the clinical part of the Rhea study (NTR 7061) to establish the diagnostic accuracy of MR-NCTI for the differentiation of acute from chronic PVT. Furthermore, the interobserver agreement of these sequences for PVT imaging will be assessed.
