# Supplementary material for: Magnetic Resonance Thrombus Imaging to Differentiate Acute from Chronic Portal Vein Thrombosis
Source: TH Open. 2020 Sep 23;4(3):e224–30. doi: 10.1055/s-0040-1716716 (PMC7511264; doi:10.1055/s-0040-1716716)
Supplement: Supplementary file 1 — Supplementary Material [file 10-1055-s-0040-1716716-s200061.pdf]

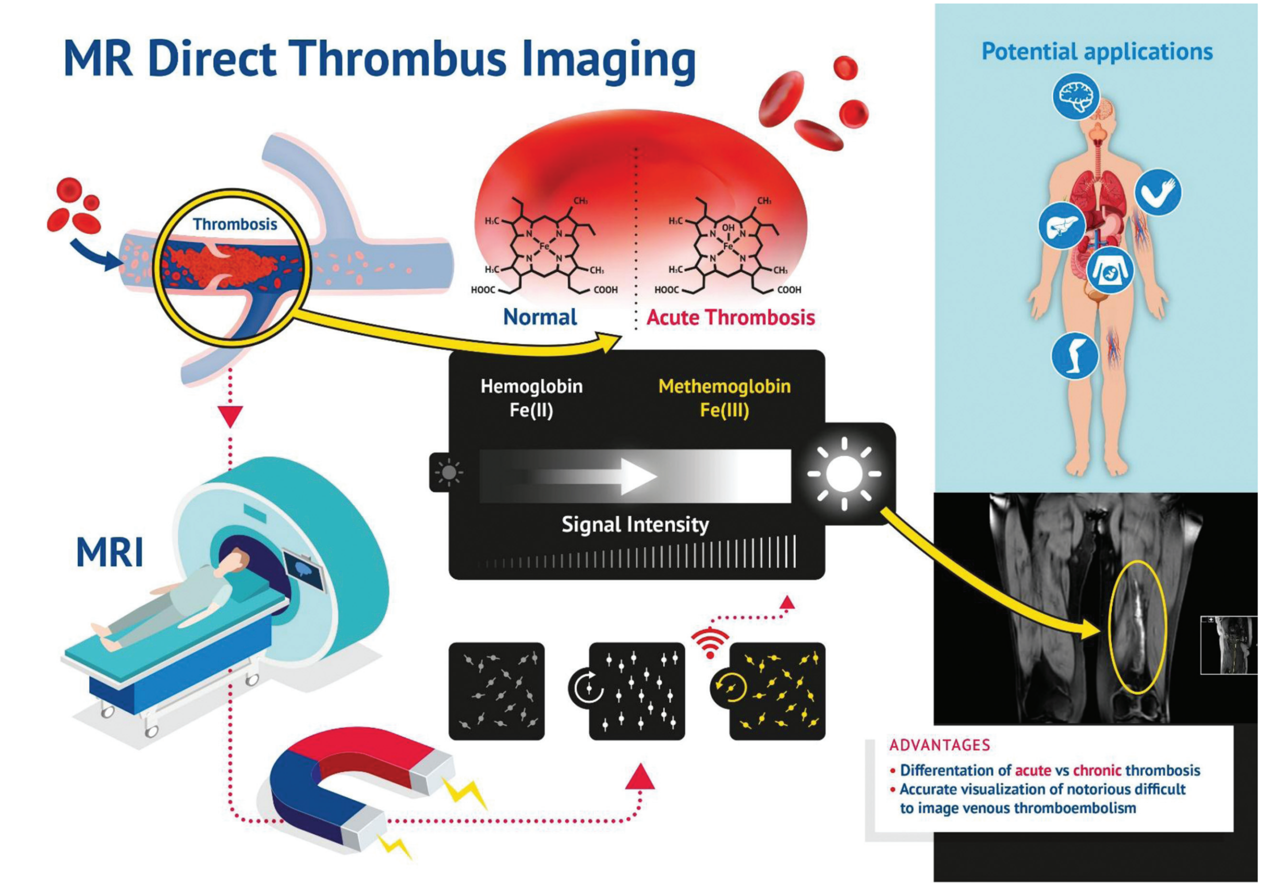

Supplementary Fig. 1 MR direct thrombus imaging or MR noncontrast thrombus imaging infographic. MR, magnetic resonance; MRI, magnetic resonance imaging. Note: This research was originally published by Van Dam et al.[9]

Supplementary Material: PubMed search string

(((“Venous Thrombosis”[mesh]) OR “splanchnic vein thrombosis”[tw] OR “splanchnic vein thromboses”[tw] OR “splanchnic venous thrombosis”[tw] OR “mesenteric vein thrombosis”[tw] OR “mesenteric vein thromboses”[tw] OR “mesenteric venous thrombosis”[tw] OR “mesenteric venous thromboses”[tw] OR “portosplenomesenteric venous thrombosis”[tw] OR “splanchnic”[ti] OR “mesenteric”[ti] OR “portosplenomesenteric”[ti]) OR “vein”[ti] OR “venous”[ti] OR (“abdominal”[ti] AND “imaging”[ti]) OR (“thrombosis”[ti] OR “thromboses”[ti] OR “embolism”[ti] OR “thrombus”[ti] OR “occlusion”[ti]))) AND (“MRDTI”[tw] OR MR-DTI[tw] OR “magnetic resonance direct thrombus imaging”[tw] OR “magnetic resonance”[tw] OR “Magnetic Resonance Imaging”[mesh] OR “Magnetic Resonance Imaging”[tw] OR “MRI”[tw] OR mr imag*[tw] OR “thrombus imaging”[tw]) OR (“Fat”[All Fields] AND “Suppression”[All Fields]) AND (“methods”[All Fields] OR “techniques”[All Fields] OR “methods”[mesh] OR “techniques”[All Fields])).
